# Melanin from the Nitrogen-Fixing Bacterium *Azotobacter chroococcum*: A Spectroscopic Characterization

**DOI:** 10.1371/journal.pone.0084574

**Published:** 2014-01-09

**Authors:** Aulie Banerjee, Subhrangshu Supakar, Raja Banerjee

**Affiliations:** Department of Bioinformatics, West Bengal University of Technology, Salt Lake, Kolkata, W.B., India; Dowling College, United States of America

## Abstract

Melanins, the ubiquitous hetero-polymer pigments found widely dispersed among various life forms, are usually dark brown/black in colour. Although melanins have variety of biological functions, including protection against ultraviolet radiation of sunlight and are used in medicine, cosmetics, extraction of melanin from the animal and plant kingdoms is not an easy task. Using complementary physicochemical techniques (*i.e.* MALDI-TOF, FTIR absorption and cross-polarization magic angle spinning solid-state ^13^C NMR), we report here the characterization of melanins extracted from the nitrogen-fixing non-virulent bacterium *Azotobacter chroococcum*, a safe viable source. Moreover, considering dihydroxyindole moiety as the main constituent, an effort is made to propose the putative molecular structure of the melanin hetero-polymer extracted from the bacterium. Characterization of the melanin obtained from *Azotobacter chroococcum* would provide an inspiration in extending research activities on these hetero-polymers and their use as protective agent against UV radiation.

## Introduction

Melanins are found widely dispersed in the animal and plant kingdoms. They have a variety of biological functions, including protection against the UV radiation of the sunlight and energy transduction [1]. Melanins influence human skin and hair colour and are found in the *medulla* and *zona reticularis* of the adrenal gland, the inner ear, and in pigment-bearing neurons within areas of the brain stem, such as the *substantia nigra*. Melanins can also protect microorganisms, such as bacteria and fungi, against thermal as well as chemical (*e.g.* heavy metals and oxidizing agents) and biochemical (*e.g.* host defenses against invading microbes) stresses [2] that involve cell damage by the solar UV radiation through generation of reactive oxygen species. A potentially novel role of melanins as photosynthetic pigments in some fungi, enabling them to capture **γ**-rays [3] and harness their energy for growth, has recently been described [4]. Organisms of the genus *Azotobacter* are free-living, non-virulent, nitrogen-fixing obligate aerobes [5]. Among various species of this genus, *Azotobacter chroococcum* has been most commonly isolated from the soils worldwide. The production of melanin by this bacterium has been reported [6]–[8]. Although the intensity of melanogenesis does not appear to be directly correlated with the nitrogenase activity, it is possible that *Azotobacter* employs melanogenesis to enhance oxygen utilization and is able to maintain the reducing conditions necessary to bind atmospheric nitrogen. The presence of iron and copper ions in the medium significantly increases the *Azotobacter* melanization process [9].

Melanins, classified as eumelanins, allomelanin, pheomelanin, pyomelanin, neuromelanin, are biosynthesized from different sources through different biochemical pathways (e.g. eumelanins from tyrosine in the presence of tyrosinase enzymes, while allomelanin from cathecol in the presence of polyphenol oxidase) [9]. These widely dispersed pigments are amorphous, heterogeneous, insoluble and resistant to crystallization. In spite of being responsible for a wide range of biological functions, this pigment has not been amenable to easy chemical and structural analyses. The poor solubility of these pigments severely limits the range of techniques useful for their investigation. However, some structural information of melanins has been derived largely from the extensive chemical degradation studies [10]–[12].

High-resolution CPMAS solid-state ^13^C NMR, along with the knowledge of established chemical shift values, have been used by several investigators for identification of the different functional groups present in synthetic as well as natural melanins derived from animal or fungal sources [13]–[16]. Signals from indolic along with aromatic, carboxylic acids and uncyclized aliphatic chains have been found in these materials, but their relative intensities differ widely in diverse sample sources [11], [13]. Although several studies pointed that dihydroxyindole moiety (dihydroxyindole and dihydroxyindole-2-carboxylic acid) act as the basic constituent of melanins (eumelanins) [10]–[12], [14], [17] and polymerize in a heteromeric distribution (the number of fundamental unit varies from three to five [14], [16]–[20]), nevertheless till today, no complete molecular structure has been reported in the literature [12], [14] for these naturally occurring hetero-polymer. However, for a few cases the empirical formula of melanin has been suggested (e.g. C_7.35_H_4.6_NO_3.6_ for *Sepia* melanin free acid (*Sepia* MFA) [21] and C_7.7_H_4.27_NO_3.32_ for the auto-oxidized DOPA melanin [22]; normalized with respect to N = 1).

As extraction of pure melanins from the animal and the plant kingdoms is not an easy task, the focus of the present study is to characterize melanins extracted from a safe and easy source: non-virulent, nitrogen-fixing bacterium *Azotobacter chroococcum*. Towards establishing the extracted compound as melanin, results from complementary physicochemical techniques have been employed to infer about the constituent functional groups of the pigment and have been compared with the available results used for characterization of different melanins. Further, on the basis of the results obtained, we have extended our effort to propose putative model structure of the constituent protomolecules for the melanins extracted from bacterium *Azotobacter chroococcum*.

## Results

The dark-brown compound(s) obtained from the bacterium *Azotobacter chroococcum* have been investigated by elemental analysis, UV-VIS, MALDI-TOF mass spectrometry, FTIR spectroscopy and solid-state ^13^C NMR spectrometry.

### I. Elemental Analysis

Presence of the nitrogen in the dark-brown compound(s) obtained has been confirmed from the Lassaigne's test (dark blue coloration) [23]. The result is further supported by the C∶H∶N analysis [carbon (47.7218%), hydrogen (2.9707%) and nitrogen (6.9024%)]. However, appearance of no coloration/precipitate in the respective Lassaigne's test may indicate the absence of sulfur and halogen. Further, as the sample is insoluble in water and does not contain sulfur and halogen, chances of interferences from other elements and ionized radicals can be ignored [24].

### II. UV-VIS spectrum

The compound(s) is soluble in 1N NaOH and shows a broad spectrum in the range of 650–200 nm (Figure S1 in File S1).

### III. Matrix-assisted laser desorption/ionization-time of flight (MALDI-TOF) analysis

**Figure 1:**
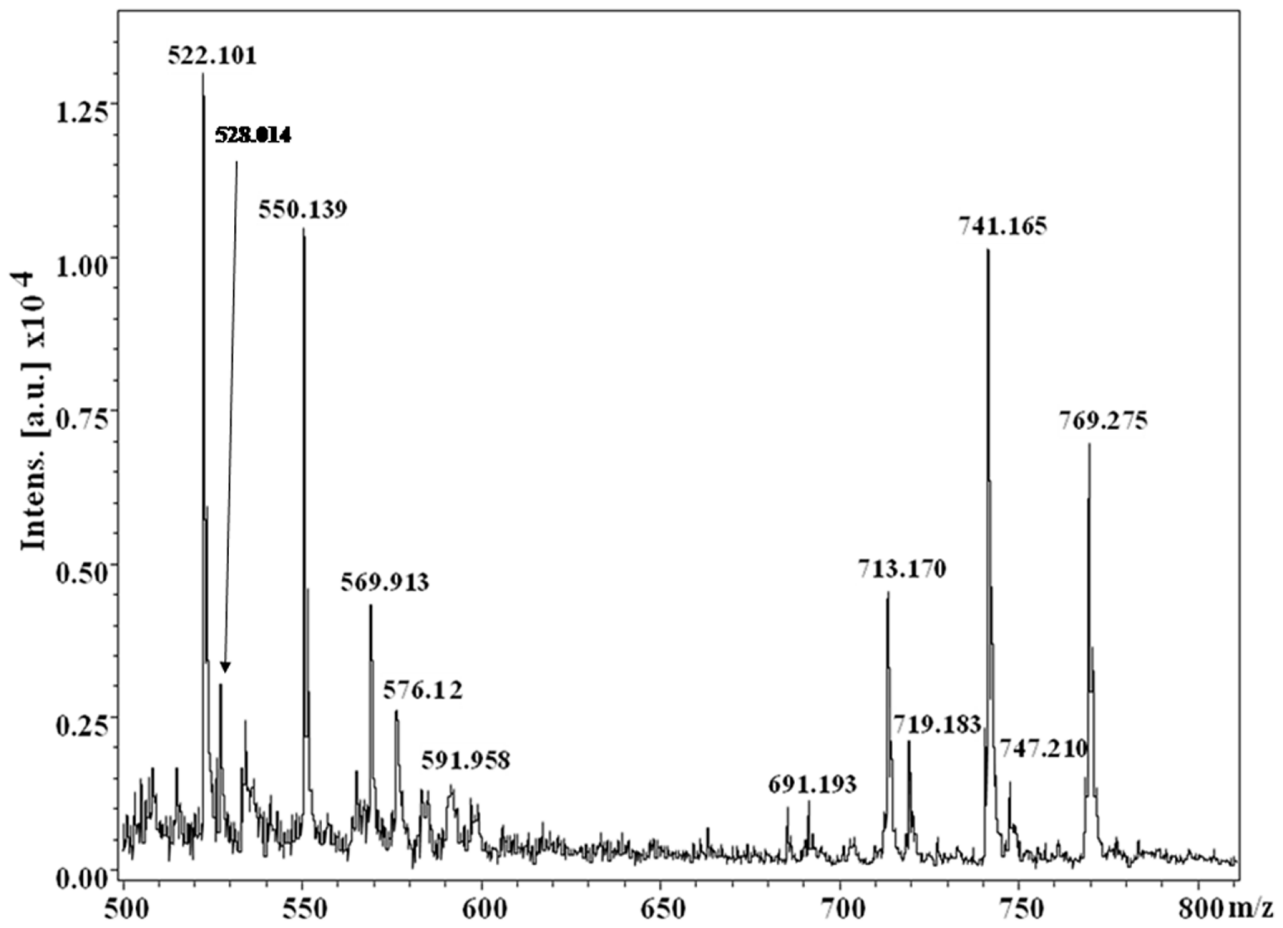
Partial MALDI-TOF MS [M+H]^+^ and [M+Na]^+^ spectrum of the dark brown compound(s) obtained from the bacterium *Azotobacter chroococcum*.

MALDI-TOF analysis of the dark-brown compound(s) demonstrates the presence of several molecular ions (from the observed m/z values) in the spectrum (*e.g.* m/z values: 522.101, 528.014, 550.139, 569.913, 576.12, 591.958, 691.193, 713.170, 719.183, 741.165, 747.210, 769.275) (Figure 1), which indicate the presence of mixture of compounds. No m/z peak has been observed beyond the value of 800. Out of these several peaks observed, m/z values corresponding to 528.014, 569.913, 691.193, 719.183 and 747.210 may be designated as [M+H]^+^ molecular ions, while the m/z values 550.139, 591.958, 713.170, 741.165 and 769.275 represent the corresponding sodiated [M+Na]^+^ species.

### IV. FTIR absorption analysis

**Figure 2:**
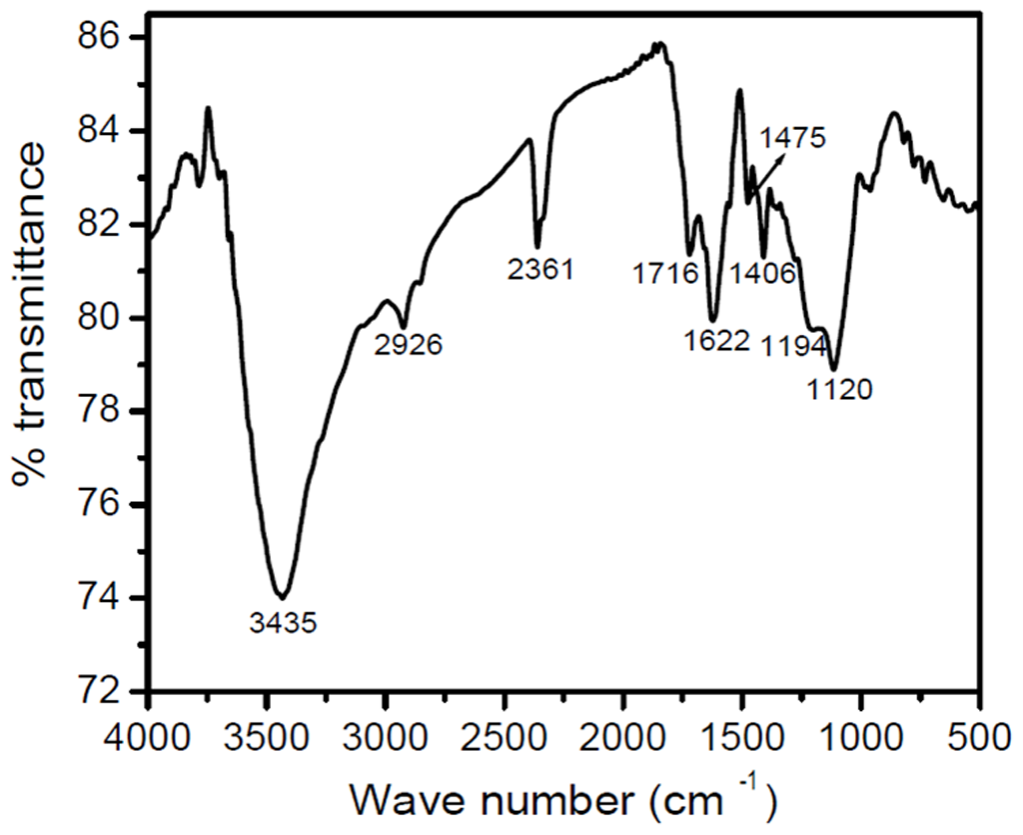
FTIR absorption spectrum (in KBr pellet under complete dry conditions in a nitrogen atmosphere) of the dark brown compound(s) obtained from *Azotobacter chroococcum*.

The FTIR absorption spectrum in KBr pellet (in complete dry conditions using a nitrogen atmosphere) shows intense peaks at 3435, 2926, 2361, 1716, 1622, 1406 and 1194 and 1120 cm^−1^ (Figure 2) which indicate the presence of several functional groups (*e.g.* C = O of -COOH, C-O of -COOH, carbonyl C = O, C = N, aromatic C = C, -OH and –NH) [25].

### V. ^13^C-NMR chemical shift analysis

**Figure 3:**
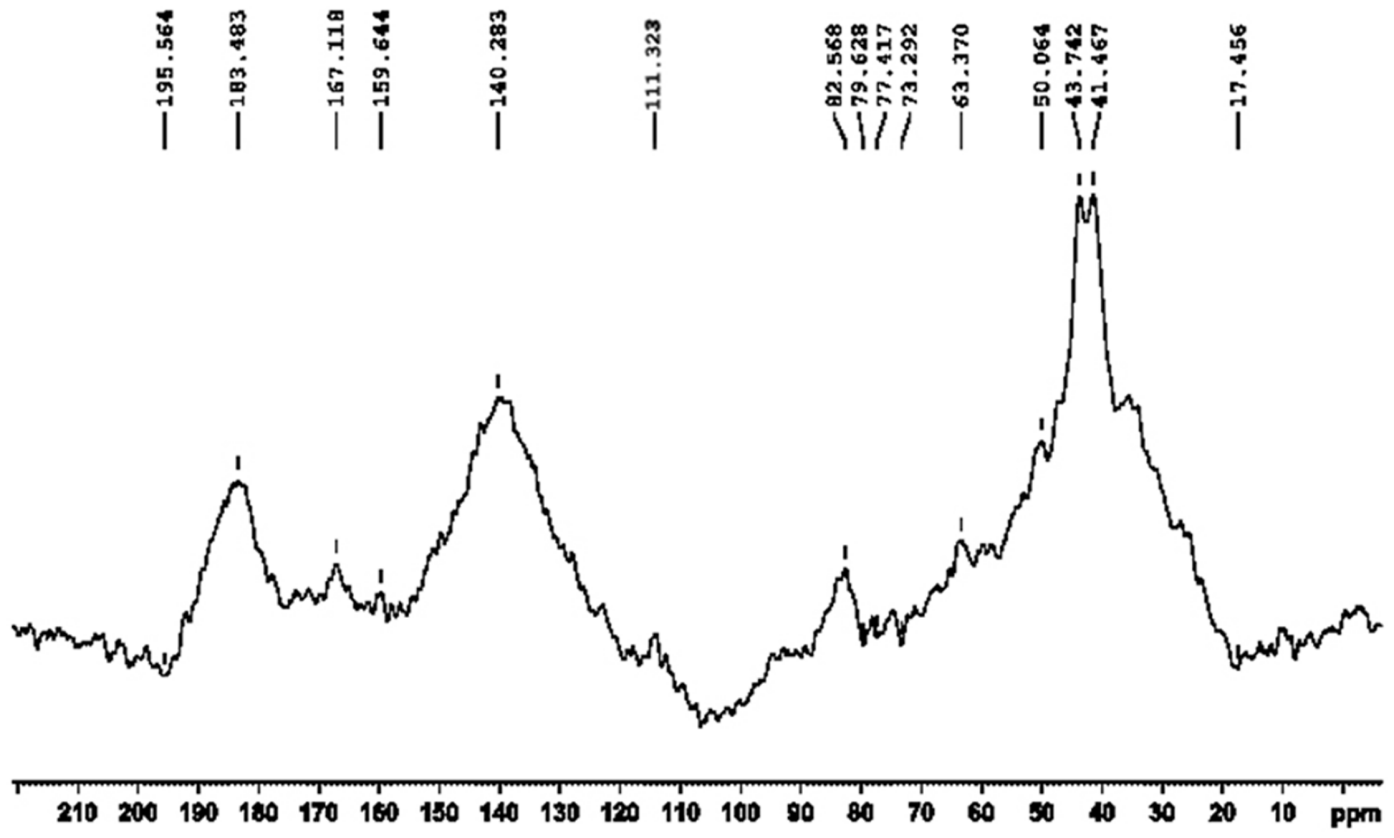
Natural abundance CPMAS solid-state ^13^C-NMR 1D-spectrum of the dark brown compound(s) obtained from bacterium *Azotobacter chroococcum*.

Cross-polarization magic-angle spinning (CPMAS) solid-state ^13^C NMR technique has been employed for further characterization, as the material is almost insoluble in water as well as in organic solvents. The overall spectrum (Figure 3) can be deconvoluted broadly into three parts: a) 160–200 ppm; b) 100–150 ppm; c) 10–90 ppm which would be attributed to carbonyl, aromatic and aliphatic carbon containing functionalities, respectively [11], [26]–[28].

## Discussion

### I. Characterization of the extracted compound as melanin

**Figure 4:**
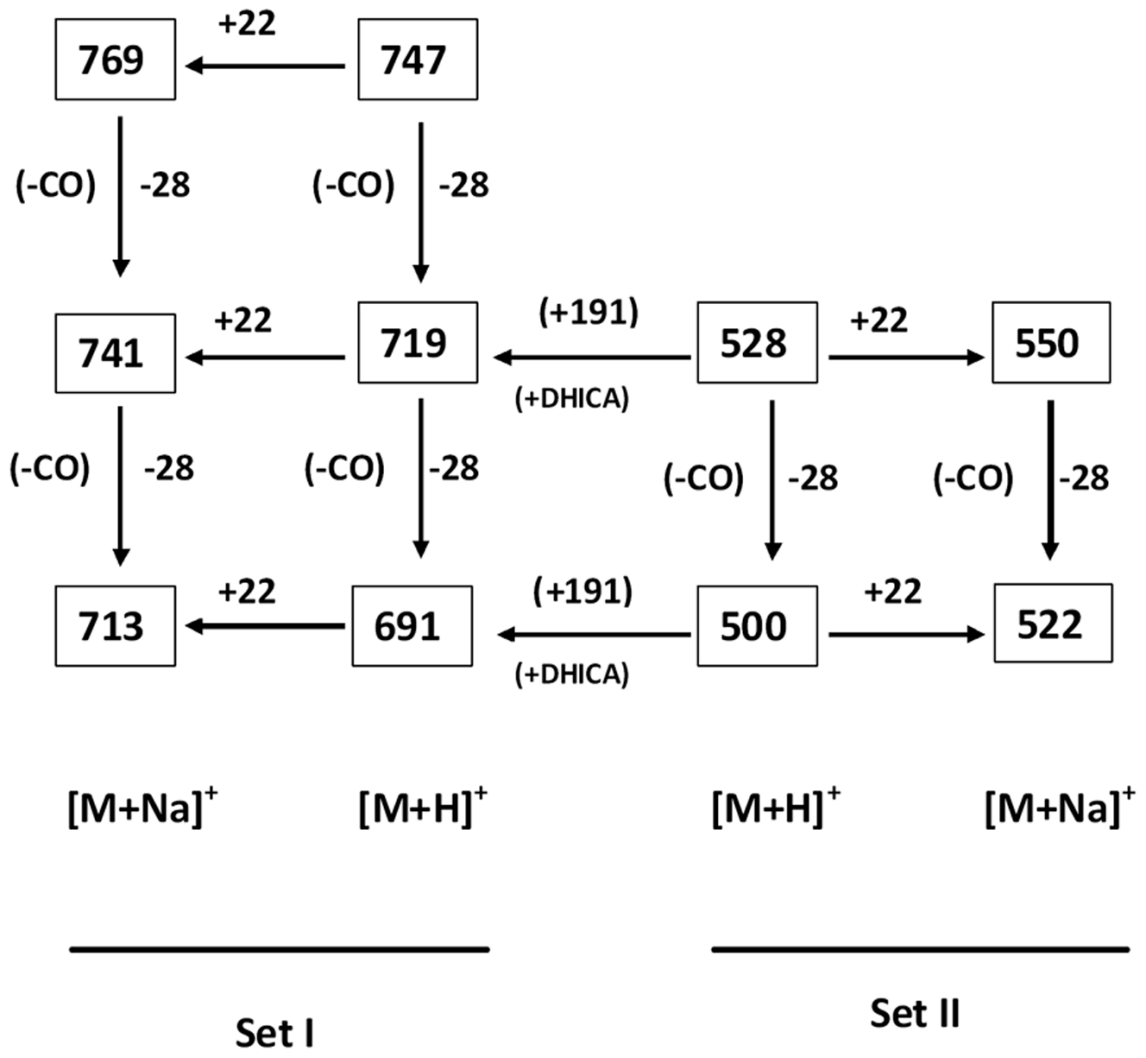
m/z values of the compound(s) obtained from the bacterium *Azotobacter chroococcum* in MALDI-TOF MS spectrum representing molecular ions corresponding to two sets of [M+H]^+^ and [M+Na]^+^ having difference of 191amu (one unit 5,6-dihydroxyindole-2-carboxylic acid).

Towards characterization of the dark brown compound(s) obtained from the bacterium *Azotobacter chroococcum*, all the attempts for purification using the HPLC technique failed. This negative result strongly indicates the presence of a polydisperse, complex heterogeneous mixture (supported by the MS spectrum and the appearance of a broad spot in TLC). However, appearance of two sets of m/z values in the range of 500 and 700 with a difference of m/z 191 (Figure 1, Figure 4) supports the view that two sets of polymers (where the number of monomeric unit varies) exists in the compounds. The possibility of existence of strongly acidic (*e.g.* -COOH) and/or weakly acidic (*e.g.* phenolic -OH group) functionality may be concluded from the solubility of the compound in 1N NaOH. The strong band at ∼3435 cm^−1^ in the FTIR spectrum can be assigned to the vibration of non-hydrogen bonded NH groups [16], [29]–[30] and the broad band observed between 3200–2000 cm^−1^ may be related to O-H stretching vibrations associated to intra/intermolecular hydrogen bonds [25]–[26]. Appearance of peaks at 2926 cm^−1^ and 1622 cm^−1^ in the FTIR spectrum can be assigned to the aromatic C-H and C = C stretching modes, respectively [25] pointing towards the presence of an aromatic system in the compound(s). This conclusion is further supported by appearance of the ^13^C peaks in the range of 110–150 ppm, corresponding to aromatic moieties. An intense peak at ∼140.3 ppm emphasizes the existence of deshielded aromatic carbon atoms, while the peak at ∼110 ppm (111.3 ppm) may be considered as the characteristic signature of indole/pyrrole carbons [31] (presence of nitrogen is confirmed through C∶H∶N analysis and elemental analysis using Lassaigne's test). M/z values having a difference of 28 in both the sets [*e.g.* for [M+H]^+^: 747, 719, 691 (for Set I) and 528, 500 (Set II) while for [M+Na]^+^: 769, 741, 713 (for Set I) and 550, 522 (for Set II)] (Figure 1, 4), may be attributed due to loss of CO. Existence of the OH functional group and the aromatic system indicated by the FTIR absorption spectrum along with the presence of aromatic/indole system as evidenced from solid state ^13^C NMR and the concomitant loss of CO from the immediate precursor observed in MS spectrum clearly indicates that the OH-functionality exists as phenolic OH group(s) in the system [32]. This signifies the presence of the hydroxyindole moiety, the basic constituent of melanins as reported by other [28], [31]. Absorbance around 320 nm in the UV-Visible spectrum supports the presence of dihydroxyindole/dihydroxyindole carboxylic acid moiety in the compound(s) and the overall broad spectrum resembles that of melanins. Appearance of a band at 1716 cm^−1^ in the FTIR absorption spectrum may be related to the stretching mode of the C = O group of carboxylic acid/ester moieties. However, this frequency value is slightly lower than that observed for a non-associated carboxylic acid [33]. This result indicates that these carboxylic acid/ester moieties may be associated with intermolecular hydrogen-bonding. The strong bands at 1195 and 1120 cm^−1^ would be related to the C–O stretching vibrations of carboxylic acids/esters and of C–OH group. The large difference between the C = O and C–O stretching frequencies of carboxylic acids support their existence as COOH group, as the IR absorption spectrum of the sodium salt of indole-2-carboxylic acid show two strong bands at 1562 and 1409 cm^−1^, assigned to the asymmetric and symmetric stretchings of the –COO^−^ group [17]. The ^13^C chemical shift value at ∼167 ppm indicates the presence of carbonyl groups of carboxylic acids similar to that observed for an indole carboxylic acid moiety [27]. Further, appreciable transmittance ∼1700–1650 cm^−1^ in the IR spectrum suggests the presence of C = O (carbonyl) group or C = N group or both, that may be associated in intra/intermolecular hydrogen bonds. Presence of strongly H-bonded secondary or tertiary amide C = O (may be due to presence of proteinaceous species) cannot be ruled out from the appearance of strong band ∼1650–1600 cm^−1^. However, appearance of the ^13^C peak at ∼159 ppm may suggest the presence of C = N group, which is probably arising from the indole/pyrrole system. Thus, the obtained IR spectra of the compound(s) under study matches very well with the solid-state FTIR absorption spectrum of the indole-2-carboxylic acid reported in literature [34] and with those of a few melanins extracted from other natural sources [35]–[38]. A closely related FTIR absorption spectrum is also obtained by us (observed peaks at: 3385, 3205, 2910, 2362, 1714, 1622, 1396, 1295 cm^−1^) for the synthetic melanin, purchased from Sigma-Aldrich (CAS No. 8049-97-6) (Figure S2 in File S1).

The ^13^C NMR spectrum obtained for the compound(s) extracted from the bacterium *Azotobacter chroococcum* resembles that of 5,6-dihydroxyindole, an important constituent of the pigment melanin [13], [16], [27], [39]. ^13^C-chemical shift value at ∼195 ppm may be associated to the carbonyl group from the quinone tautomer of the dihydroxyindole compound. This hypothesis is inferred from the theoretically calculated chemical shift values of different tautomers of 5,6-dihydroxyindole and 5,6-dihydroxyindolecarboxylic acid using the ChemBioDraw Ultra 12.0 (Figure S3 in File S1). The appearance of peaks at ∼10–80 ppm (in particular intense peak between 50 and 35 ppm) establishes the presence of aliphatic carbon atoms and matches well with the chemical shift values of aliphatic carbons of several model compounds (L-dopa, dopamine, 2-methoxycarbonyl-3-ethoxycarbonyl-4-methylpyrrole,ethyl5,5-dimethoxyindole-2-arboxylate etc.) used for the elucidation of melanin structure [27]–[28]. The overall ^13^C spectrum obtained from the compound(s) under investigation is very similar to that of the melanins obtained from *Sepia* melanin, human hair melanin, dopa melanin and melanoma melanin (Table 1) [27]–[28].

**Table 1:** Comparison of the ^13^C resonances of the compound(s) (melanin) extracted from bacterium *Azotobacter chroococcum* with other type of melanins obtained from different sources [^a^ Magn. Reson Chem. (2008) *46*, 471; ^b^ Magn. Reson Chem. (2003) *41*, 466].

Compounds	^13^C resonances		
	Carbonyl	Aromatic	Aliphatic
Dopa melanin^a^	172	143-118	35
Melanoma melanin^b^	173	125	53,33
Sepia melanin^b^	200-160	150-110	90-30
Sepia Melanin Free Acid^b^	200-160	150-110	90-30
Human hair melanin^b^	200-170	135-110	90-30
Compound(s) from *A. chroococcum*	200-160	150-115	90-25

Moreover, the ^13^C NMR spectrum of *Sepia* melanin, in the region from 80 to 20 ppm, shows a broad resonance due to many overlapping peaks. Similar spectrum is also observed for the compound(s) under study. Absence of peak at ∼90–105 ppm found in the spectrum of the compound under study (reported for the model compound 5,5-dimethoxyindole-2-carboxylate [26]) indicates substitutions at the aromatic carbons, emphasizing co/hetero polymerization at the indole moiety. Such substitutions found in the aromatic carbons of *Sepia* melanin and human hair melanin is responsible for the formation of the polymeric structures of the respective melanins. The broad features of the observed spectrum, similar to those of *Sepia* melanin and human hair melanin, may indicate heterogeneity in the polymer, a well-known aspect of melanin structure, as well as the presence of free radicals. Moreover, the poor signal-to-noise ratio in the spectrum reported in Figure 3 may be the result of dipolar line broadening due to the presence of unpaired electrons, as found in *Sepia* melanin and noted in an EPR study [40].

As the overall FTIR absorption and ^13^C NMR spectra emphasize the occurrence of a hydroxy-indolecarboxylic acid moiety in the extracted compound(s), one can conclude that 5,6-dihydroxyindole-2-carboxylic acid along with its tautomeric form, reported as the main constituents of melanins [10], [12], represents the basic units of the dark brown compound(s) extracted from the bacterium *Azotobacter chroococcum*. In addition, as the extracted compound(s) is chemically similar to the melanins obtained from several different sources; using the complementary techniques one can unambiguously establish that the dark brown compound(s) extracted from *Azotobacter chroococcum* would be none other than melanins, constituted by the hydroxyindole moiety, as reported for eumelanins.

Finally, presence of the proteinaceous material in the compound seems to be a logical conclusion from the appearance of peaks at ∼165–200 ppm in the ^13^C NMR spectrum along with the appearance of strong band ∼1650–1600 cm^−1^ in the FTIR absorption spectrum (due to amide carbonyls). However, from the almost comparable peak intensity ratio of the aliphatic to aromatic signals obtained in the ^13^C spectrum, one can justify that the presence of the proteinaceous material, if any, is of minor significance. This conclusion can be validated from the literature survey of the ^13^C CPMAS studies of the *Sepia* melanin and human hair melanin, which shows that the intensity ratio of aliphatic to aromatic signals for *Sepia* melanin is comparable (amino acids account for 6.17%) while that for human hair melanin is substantially larger (amino acids account for 66.8%) [27].

### II. Towards model structure(s)

Melanins are considered to be heteropolymers constituted of the indole moieties and linked via carbocycles or heterocycles, predominantly polymerized through C-C linkages [12], [41]. Nevertheless, so far no molecular structure of melanin has been proposed, as the molecular weight of this heteropolymer was not obtained with a reliable accuracy. The dark brown compound(s) melanins, extracted from the bacterium *Azotobacter chroococcum*, contain nitrogen but neither sulfur nor halogen. Difference of m/z of 191amu between set-I and set-II (Figure 1, 4) of the extracted melanin in MALDI-TOF experiment, a very good tool for ascertaining the molecular weight of the compound(s), can be attributed to single unit of 5,6-dihydroxyindole-2-carboxylic acid (DHICA), recognized as an important constituent of melanins (eumelanin). These results would clearly corroborate and justify that DHICA would act as the basic constituent of the heteropolymer (melanins) under study. Further, the observed m/z values are quite similar to the m/z values (e.g. 524, 552, 576, 598, 698, 767, 787) obtained from the chemical and enzymatic oxidations of 5,6-dihydroxyindole-2-carboxylic acid (DHICA) using the MALDI-TOF technique [42].

From the complementary physicochemical studies, it can be reasonably concluded that, like other melanins reported in literature, 5,6-dihydroxyindole and/or 5,6-dihydroxyindole-2-carboxylic acid/ester would be the constituent monomeric units for the melanins extracted from the bacterium *Azotobacter chroococcum* and the compound is under the category of eumelanins although obtained from a bacterium, which may be a strain related phenomenon. However, recent studies on production of melanin revealed that in some cases even bacteria can produce eumelanins [43]–[44].

**Figure 5:**
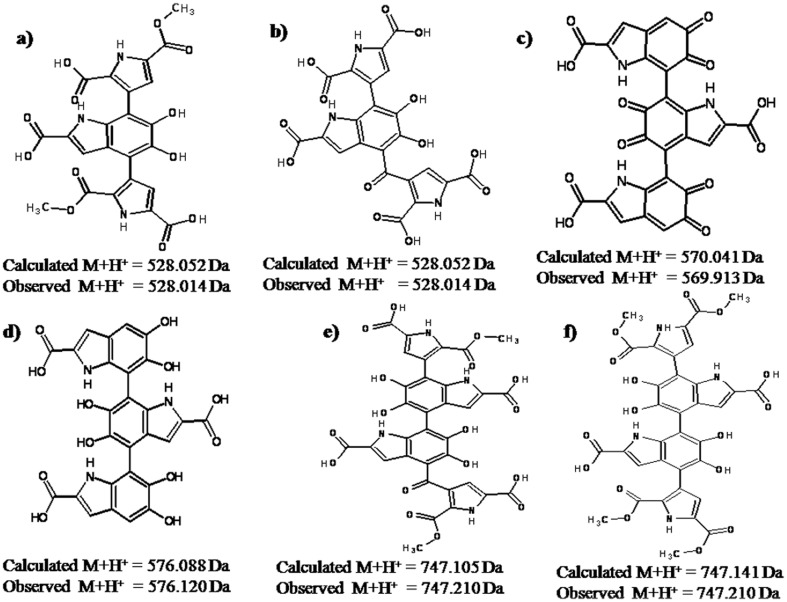
Proposed putative structure(s) of the protomolecules of melanin hetero-polymers, obtained from the nitrogen-fixing soil bacterium *Azotobacter chroococcum*.

At this point, on the basis of the information obtained from various complementary spectroscopic techniques described above, we are extending an effort to propose a putative model structure for melanins protomolecules obtained from *Azotobacter chroococcum* having m/z values of [M+H]^+^ : 528.014, 569.913, 576.12, 747.210 respectively [as the others are generated from loss of CO from their immediate precursor (Figure 4)] (Figure 5). These proposed model structures (Figure 5) would be well justified and validated by their respective calculated m/z values [M+H]^+^ as well as by the theoretically predicted/calculated chemical shift values from ^13^C NMR spectroscopy using the Chem Ultra software (Figure S4 in File S1). However, it should be stated that the putative structures of melanins proposed here may not exactly match with those of the naturally occurring hetero-polymers, as there may be partial degradation/oxidation of these polydisperse compound(s) during the extraction process. Characterization of melanins obtained from a nitrogen-fixing, non-virulent bacterium *Azotobacter chroococcum*, thus leads to a safe and easy source for this photo-protective pigment. These results would allow expansion of experimental studies on melanins as protective agent against UV radiation and development of novel ways to administer this pigment in hypo- as well as hyper-pigmentation.

## Methods

The soil sample was collected from a farmland at Howrah, West Bengal, India (22°35′24″N and 88°18′36″E), dried and pulverized aseptically. The farmland is the family/ancestral property of author Ms Aulie Banerjee and as a family member she, one of the owners of the land, would not require any permission for sample collection from her own land. The species used here is not a protected or endangered one. 10 gm were shaken in 90 ml sterile distilled water for 15 min. 1 ml of the suspension was diluted in 9 ml of 1% mannitol and 1 ml of it was plated onto the Burk nitrogen free agar medium [45]–[47] and was allowed to grow for 7 days at 30°C. A few dark black/brown spots were observed measuring around 1.5 cm in diameter. The isolates were purified by streaking on Petri plates and the purified isolates were grown in liquid media [48]. The media (pH 7) was kept at 30°C. Based on the observations of the colony morphology and coloration, cell nature, mean dimensions of the cells along with the flagella pattern and the pigment produced, the strain was identified and characterized [5], [49].

### Extraction of melanins

Isolation of melanin from the cells of *Azotobacter chroococcum* through the protocol described here has been reported earlier [7]. The culture was centrifuged at 1000 g for 5 min to pallet the cells. The cell pallet was extracted three times with 5% trichloroacetic acid, washed twice with ether-ethanol (1∶1 volume/volume) then washed once with absolute ether to remove impurities. The residual material was then dissolved in 0.05M sodium carbonate by treatment in a 100°C water bath for 10 min. Further centrifuge the solution to remove insoluble material. After that the mixture was stored and suspended at room temperature for 15 minutes. The brown-black material was washed three times with deionised water and freeze dried to obtain a brown-black powder which is used for further experiments.

### MALDI-TOF

MALDI-TOF experiment was performed as positive mode in a Bruker Daltonics Autoflex TOF/TOF instrument. α-cyano-4-hydroxycinnamic acid was used as the matrix and the Flex Analysis software was used for analyzing the [M+H]^+^ results.

### FTIR absorption spectroscopy

Melanin powder (obtained from the bacterium) and KBr (purchased from Sigma) were mixed in a 1∶100 w/w. The mixture was ground using a mortar pestle till it achieved a uniform color indicating its homogeneity. FTIR absorption spectrum was obtained at 25°C using a model Bx Perkin-Elmer FTIR spectrophotometer Spectrum 1000, using 4 cm^−1^ resolution with 8 number of scans.

### CPMAS ^13^C NMR spectrometry

The NMR data were recorded on a Bruker DSX 300, 7.04 Tesla, solid-state NMR spectrometer using 5-mm probes. For the ^13^C CPMAS experiments, >100 mg of sample were tightly packed using 4-mm rotors with teflon spacers and spun at a typical speed of 1.2 kHz. ^13^C CPMAS experiments were conducted with a ^1^H decoupling strength of 50 kHz, a delay time of 1 s between successive acquisitions, a line broadening of 50–100 Hz, and contact times of 2 ms to establish the ratios of rigid carbon moieties [50]. Chemical shift referencing of ^13^C NMR studies was performed by setting the glycine -CO- at 176 ppm in a separate experiment using a pure glycine sample.

## Supporting Information

File S1 **Supporting figures.** Figure S1, UV-VIS absorption spectrum of the dark brown compound(s) obtained from *Azotobacter chroococcum* in NaOH. Figure S2, FTIR spectrum (in KBr pellet under complete dry condition in nitrogen atmosphere) obtained from synthetic melanin purchased from SIGMA ALDRICH. Figure S3, a) Calculated ^13^C-NMR chemical shift values using the Chem Ultra software for 5,6-dihydroxyindole and its tautomer b) Calculated ^13^C-NMR chemical shift values using the Chem Ultra software for 5,6-dihydroxyindole-2-carboxylic acid and its tautomer, Figure S4, Calculated ^13^C-NMR chemical shift values using the Chem Ultra software for the proposed putative structure(s) of the protomolecules of melanin hetero-polymers obtained from the nitrogen-fixing soil bacterium *Azotobacter chroococcum*.
(DOC)
