# Supplementary material for: Melanin from the Nitrogen-Fixing Bacterium Azotobacter chroococcum: A Spectroscopic Characterization
Source: PLoS One. 2014 Jan 9;9(1):e84574. doi: 10.1371/journal.pone.0084574 (PMC3887007; doi:10.1371/journal.pone.0084574)
Supplement: File S1 — Supporting figures. Figure S1, UV-VIS absorption spectrum of the dark brown compound(s) obtained from Azotobacter chroococcum in NaOH. Figure S2, FTIR spectrum (in KBr pellet under complete dry condition in nitrogen atmosphere) obtained from synthetic melanin purchased from SIGMA ALDRICH. Figure S3, a) Calculated 13C-NMR chemical shift values using the Chem Ultra software for 5,6-dihydroxyindole and its tautomer b) Calculated 13C-NMR chemical shift values using the Chem Ultra software for 5,6-dihydroxyindole-2-carboxylic acid and its tautomer, Figure S4, Calculated 13C-NMR chemical shift values using the Chem Ultra software for the proposed putative structure(s) of the protomolecules of melanin hetero-polymers obtained from the nitrogen-fixing soil bacterium Azotobacter chroococcum. (DOC) [file pone.0084574.s001.doc]

**Supporting Information**

**Melanin from the Nitrogen-fixing Bacterium *Azotobacter chroococcum:* A spectroscopic characterization**

**Aulie Banerjee, Subhrangshu Supakar and Raja Banerjee***

Department of Bioinformatics, West Bengal University of Technology, BF-142,

Salt Lake, Kolkata, W.B., India

Telephone No: 91-33-23341021/1031 (Extn. 146); Fax: 91-33-2334-1030

Email:  [ban_raja@yahoo.com, banraja10@gmail.com](mailto:ban_raja@yahoo.com)

____________________

* Correspondence to: Dr. Raja Banerjee, Department of Bioinformatics, West Bengal University of Technology, BF-142, Sector-1, Salt Lake, Kolkata- 700064, India.

E-mail: ban_raja@yahoo.com

**Supporting Figures**

**Figure** S**1:** UV-VIS absorption spectrum of the dark brown compound(s) obtained from *Azotobacter chroococcum* in NaOH.

**Figure S2:** FTIR spectrum (in KBr pellet under complete dry condition in nitrogen atmosphere) obtained from synthetic melanin purchased from SIGMA ALDRICH.

**a)**

Molecular Formula = C_8_H_7_NO_2_ Molecular Formula = C_8_H_5_NO_2_

% of C, H, N, O = % of C, H, N, O =

C(64.42%) H(4.73%) N(9.39%) O(21.45%) C(65.31%) H(3.43%) N(9.52%) O(21.75%)

Calculated [M+H]+ = 150.054955 Da Calculated [M+H]+ = 150.054955 Da

**b)**

Molecular Formula = C_9_H_7_NO_4_ Molecular Formula = C_9_H_5_NO_4_

% of C, H, N, O = % of C, H, N, O =

C(55.96%) H(3.65%) N(7.25%) O(33.13%) C(56.55%) H(2.64%) N(7.33%) O(33.48%)

Calculated [M+H]+ = 194.044784 Da Calculated [M+H]+ = 194.044784 Da

**Figure** **S3: a)** Calculated ^13^C-NMR chemical shift values using the Chem Ultra software for 5,6-dihydroxyindole and its tautomer

**b)** Calculated ^13^C-NMR chemical shift values using the Chem Ultra software for 5,6-dihydroxyindole-2-carboxylic acid and its tautomer

**Figure** **S4:** Calculated ^13^C-NMR chemical shift values using the Chem Ultra software for the proposed putative structure(s) of the protomolecules of melanin hetero-polymers obtained from the nitrogen-fixing soil bacterium *Azotobacter chroococcum*

a)

Calculated M+H+ = 528.052 Da

Observed M+H+ = 528.014 Da

Composition = C(52.38%) H(3.25%) N(7.97%) O(36.40%)

Molecular Formula = C_23_H_17_N_3_O_12_

Empirical Formula = C_7.67_H_5.67_NO_4_

Contd..

b)

Calculated M+H+ = 528.052 Da

Observed M+H+ = 528.014 Da

Composition = C(54.70%) H(3.51%) N(7.50%) O(34.29%)

Molecular Formula = C_22_H_13_N_3_O_13_

Empirical Formula = C_7.33_H_4.33_NO_4.33_

Contd..

c)

Calculated M+H+ = 570.041 Da

Observed M+H+ = 569.913 Da

Composition = C(56.95%) H(1.95%) N(7.38%) O(33.72%)

Molecular Formula = C_27_H_11_N_3_O_12_

Empirical Formula = C_9_H_3.67_NO_4_

Contd..

d)

Calculated M+H+ = 576.088 Da

Observed M+H+ = 576.120 Da

Composition = C(56.36%) H(2.98%) N(7.30%) O(33.36%)

Molecular Formula = C_27_H_17_N_3_O_12_

Empirical Formula = C_9_H_5.67_NO_4_

Contd..

e)

Calculated M+H+ = 747.105 Da

Observed M+H+ = 747.210 Da

Composition = C(53.09%) H(2.97%) N(7.50%) O(36.43%)

Molecular Formula = C_33_H_22_N_4_O_17_

Empirical Formula = C_8.25_H_5.5_NO_4.25_

Contd…

f)

Calculated M+H+ = 747.141 Da

Observed M+H+ = 747.210 Da

Composition = C(54.70%) H(3.51%) N(7.50%) O(34.29%)

Molecular Formula = C_34_H_26_N_4_O_16_

Empirical Formula = C_8.5_H_6.5_NO_4_
